# Discovery of tear biomarkers in children with chronic non-infectious anterior uveitis: a pilot study

**DOI:** 10.1186/s12348-018-0156-5

**Published:** 2018-10-16

**Authors:** Sheila T. Angeles-Han, Steven Yeh, Purnima Patel, Duc Duong, Kirsten Jenkins, Kelly A. Rouster-Stevens, Mekibib Altaye, Ndate Fall, Sherry Thornton, Sampath Prahalad, Gary N. Holland

**Affiliations:** 1 Division of Rheumatology, Cincinnati Children’s Hospital Medical Center, 3333 Burnet Avenue, MLC 4010, Cincinnati, OH 45229 USA; 2 Department of Pediatrics, University of Cincinnati, Cincinnati, OH USA; 3 Department of Ophthalmology, Emory Eye Center, Emory University School of Medicine, 1365 Clifton Rd B, Atlanta, GA 30322 USA; 4 Emory Integrated Proteomics Core, Emory University, 1510 Clifton Rd, Atlanta, GA 30322 USA; 5 Childrens Healthcare of Atlanta, Emory Children’s Center, 2015 Uppergate Drive, Atlanta, GA 30322 USA; 6 Department of Pediatrics, Emory University School of Medicine, Atlanta, GA USA; 7 Department of Human Genetics, Emory University School of Medicine, Atlanta, GA USA; 8 Division of Biostatistics and Epidemiology, Cincinnati Children’s Hospital Medical Center, University of Cincinnati, 3333 Burnet Avenue, MLC 5041, Cincinnati, OH 45229 USA; 9 UCLA Stein Eye Institute and David Geffen School of Medicine at University of California, 100 Stein Plaza, Los Angeles, CA 90095-7000 USA

**Keywords:** Juvenile idiopathic arthritis, Uveitis, Biomarkers

## Abstract

**Background:**

Biomarkers in easily obtained specimens that accurately predict uveitis in children with juvenile idiopathic arthritis (JIA) are needed. Aqueous humor has been studied for biomarkers, but is not routinely available. We evaluated tears from children with chronic anterior uveitis (CAU) for biomarkers reported in aqueous humor.

In this pilot study, we used Schirmer strips to collect tears from seven children (nine eyes); three children had JIA- associated uveitis (JIA-U) and four had idiopathic disease (I-CAU). Liquid chromatography-tandem mass spectrometry was used to identify and quantify tear proteins. The Mann-Whitney *U* test identified differential tear protein expression between children with JIA-U and those with I-CAU.

**Results:**

S100A9, LAP3, TTR, MIF, sCD14, S100A8, and SAA1 were detected in tears of all children; the same cytokines have been reported in aqueous humor of children with JIA-U. Tears from children with JIA-U had higher expression of proteins associated with inflammatory arthritis (SEMA3G, TIMP1, HEXB, ERN1, and SAA1) than tears from those with I-CAU. In addition, we found higher expression of sCD14, S100A8, and SAA1, but lower expression of S100A9, LAP3, TTR, and MIF, in tears from children with JIA-U compared to tears from those with I-CAU.

**Conclusions:**

Tears contain similar cytokine profiles to aqueous humor in children with CAU and may be a clinically useful source of disease biomarkers. Tears from children with JIA-U also contain cytokines associated with inflammatory arthritis; furthermore, differential expression of other tear proteins as well may provide clues to intrinsic differences between JIA-U and I-CAU, despite their similar clinical phenotypes.

**Electronic supplementary material:**

The online version of this article (10.1186/s12348-018-0156-5) contains supplementary material, which is available to authorized users.

## Background

Chronic anterior uveitis is the most common extra-articu- lar manifestation of juvenile idiopathic arthritis (JIA), oc- curring in 20% of affected children [1–4]. Of note is the fact that, in 10% of all cases, anterior uveitis develops prior to arthritis [1]. Idiopathic chronic anterior uveitis (I-CAU) and JIA-associated uveitis (JIA-U) have similar clinical phenotypes, although inflammatory arthritis is not present in children with I-CAU. Understanding the underlying pathogenesis of both inflammatory arthritis and uveitis may have diagnostic and treatment implications in childr- en with JIA.

JIA-U is typically asymptomatic, and routine ophthal- mology screening is recommended [5]. Children at highest risk for uveitis are those with oligoarticular and polyarticular rheumatoid factor (RF)-negative JIA, who are < 7 years old at JIA onset, with < 4 years duration of JIA, and who are antinuclear antibody (ANA)-positive. These factors are not, however, adequate to stratify risk accurately [6, 7]. Serum and aqueous humor (AqH) have shown differential expression of cytokines and chemokines (e.g., interleukin-29/interferon-λ1 [IL-29/IFN-λ1], transthyretin [TTR]) in children with JIA-U [8–12]; however, the invasive nature of AqH collection precludes collection from children with JIA but no uveitis and from those who are not undergoing eye surgery. Serum also may not accurately reflect ocular inflammation [8, 9]. Tears are used in biomarker studies of rheumatoid arthritis (RA), Sjögren’s disease, and other systemic diseases [13–15]. Analysis of tears may also be a non-invasive approach to assess biomarkers of uveitis, in order to identify children with JIA who are most susceptible to uveitis, and it may be reflective of intraocular pathology. Differences in the levels of cytokines and chemokines have been reported in the tears of adults with uveitis compared to healthy subjects [16].

Our objectives were to use tears to (1) determine if cytokines and chemokines reported to be present in AqH of children with uveitis are observed in tears and (2) assess the comparative tear proteomic milieu in children with JIA-U and I-CAU.

## Results

### Characteristics of children

Nine affected eyes of seven children (two oligoarticular and one polyarticular RF negative JIA-U and 4 I-CAU) were evaluated (Table 1). LC-MS/MS/SPS-MS3 was used to determine proteomic profiles from tears collected using Schirmer strips. We quantified 1804 unique proteins, following a QC step that filtered proteins based on expression levels, and 1605 proteins had a present call in half of the samples. These 1605 proteins were used in subsequent analysis.

**Table 1:** Demographics and clinical characteristics of children

*N* (%) unless otherwise specified	All *N* =7 9 eyes	JIA-U *N* =3 4 eyes	I-CAU *N* =4 5 eyes
Female	6 (86)	3 (100)	3 (75)
Race			
Caucasian	3 (43)	2 (67)	1 (25)
African-American	4 (57)	1 (33)	3 (75)
Non-Hispanic	7 (100)	3 (100)	4 (100)
Disease characteristics			
Median age at uveitis diagnosis, years, SD	6, 9	6, 5.6	8.8, 5.3
Median age at tear collection, years, SD	15, 1.8	–	–
Bilateral disease	6 (86)	2 (67)	4 (100)
Ocular complications^a^	7 (100)	3 (100)	4 (100)
ANA-positive	4 (57)	3 (100)	1 (25)
Medications			
Topical glucocorticoids	4 (57)	1 (33)	3 (75)
Topical glaucoma medications	1 (14)	0 (0)	1 (25)
Methotrexate	4 (57)	1 (33)	3 (75)
Mycophenolate mofetil	1 (14)	0 (0)	1 (25)
Infliximab	1 (14)	1 (33)	0 (0)
Adalimumab	2 (28)	1 (33)	1 (25)

^a^Ocular complications: synechiae, cataracts, glaucoma, ocular hypertension, band keratopathy, cystoid and macular edema

**Table 2:** Cytokines detected in tears of JIA-U and I-CAU in this study and from aqueous humor in previous studies

Authors, year	Disease ^a^ (*n*)	Samples	Cytokines/chemokines	Direction	Present findings JIA-U vs. I-CAU
Haasnoot, 2016 [9]	JIA-U [21] CAU [15] IU [28] Controls [8]	AqH	IL-29/ IFN-λ1	Decreased in JIA-U vs CAU, IU and controls	Not detected
			LAP	Increased in JIA-U vs IU and controls	Decreased
			S100A8	Decreased JIA-U vs. IU	Increased
			sCD14	Increased JIA-U vs. controls	Increased
Walscheid, 2015 [12]	JIA-U [17] IAU [12] Controls [16]	AqH	S100A8	Increased JIA-U and IAU vs. controls	Increased
			S100A9	Increased JIA-U and IAU vs. controls	Decreased
Ayuso, 2013 [8]	JIA-U [14] CAU [8] Other Uveitis [30] Controls [20]	AqH	TTR	Increased JIA-U and CAU vs. other uveitis and controls	Increased

*JIA-U* JIA-associated uveitis, *CAU* chronic anterior uveitis, *IU* idiopathic uveitis which included non-anterior uveitis, *IAU* idiopathic anterior uveitis, *IL-29/IFN-*λ*1*

interleukin-29/interferon-λ1, *LAP* latency associated peptide, *S100* S100 calcium binding protein, *sCD14* soluble cluster of differentiation 14, *TTR* transthyretin

^a^Disease categories are listed are as they were presented in each study and do not necessarily conform to our disease descriptions

### Cytokines/chemokines in tears

**Fig. 1:**
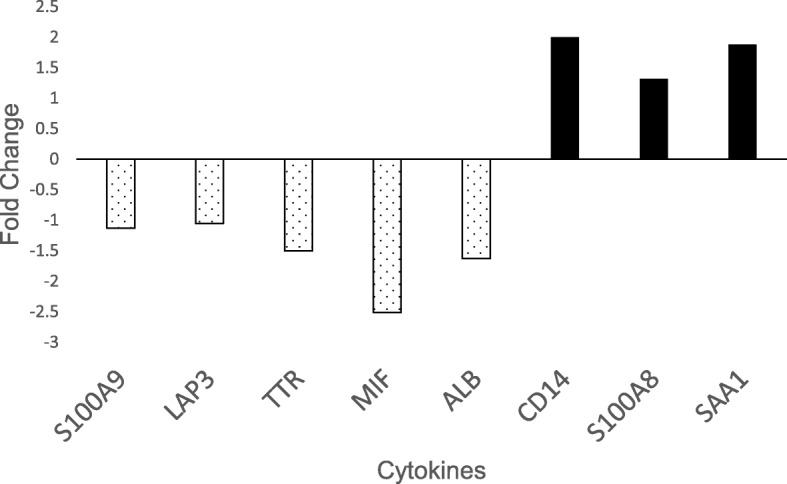
Differences in expression level (fold change) of seven cytokines of interest from earlier aqueous humor and serum studies [8–12] in patients with JIA-U compared to those with I-CAU. Black bars depict proteins that are upregulated, and stippled bars show proteins that are downregulated

We first performed a targeted analysis for cytokines and chemokines reported in AqH (Additional file 1) [8–11]. We detected CD14, S100 calcium binding protein (S100) A8/A9, serum amyloid A (SAA1), latency-associated peptide (LAP3), TTR, and macrophage migration inhibitory factor (MIF) in all nine tear samples. On further comparison, CD14, S100A8, and SAA1 had higher expression in children with JIA-U compared to I-CAU, while S100A9, LAP3, TTR, and MIF had lower expression in children with JIA-U (Fig. 1).

### Tear proteins in children differentially detected between JIA-U and I-CAU

**Fig. 2:**
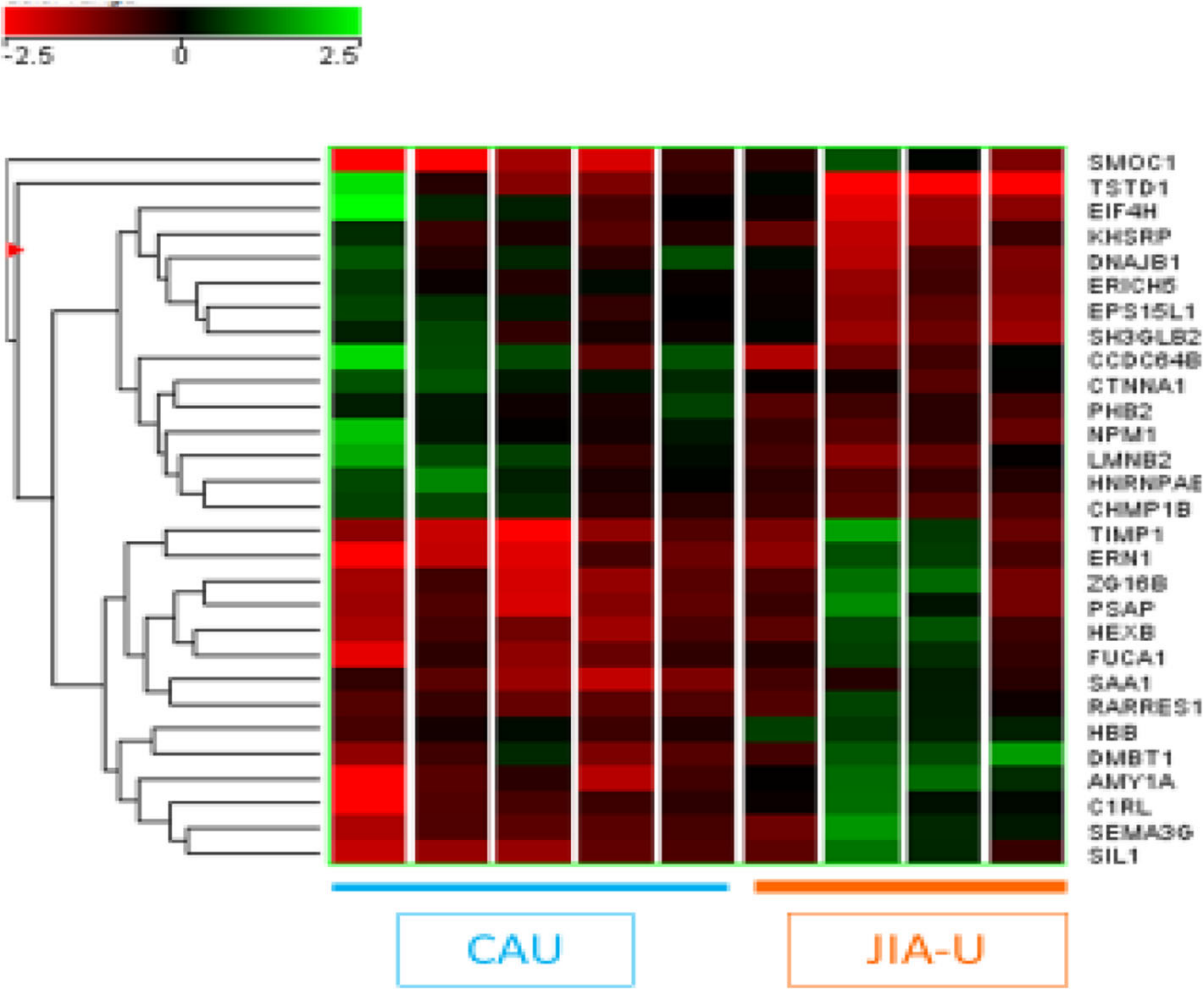
Cluster analysis of 29 proteins differentially expressed between patients with JIA-U (orange) and I-CAU (blue). The list of differentially expressed proteins was generated using Mann-Whitney *U* test, with *p* < 0.05. Complete linkage clustering algorithm, in which distance is a measure of similarity, was used to generate the hierarchical clustering tree. In the tree, each row represents a separate protein and each column represents a sample. The normalized expression level of each protein (rows) in each sample (columns) is indicated by color. Green, black, and red reflect high, medium, and low expression, respectively

We identified 29 unique proteins with significant differences between JIA-U and I-CAU. We then performed hierarchical clustering analysis using these differentially expressed proteins (Fig. 2). Green, black, and red reflect high, average, and low expression, respectively, in JIA-U compared to I-CAU. As shown in Fig. 2, these proteins could differentiate between samples obtained from children with JIA-U and I-CAU. Fourteen proteins were expressed at a higher level in JIA-U, and 15 were expressed at a lower level in JIA-U compared to I-CAU. Proteins with biological relevance were SAA1, metalloproteinase inhibitor 1 (TIMP1), beta-hexosaminidase subunit beta (HEXB), and serine/threonine-protein kinase/endoribonuclease IRE1 (ERN1), which were increased in JIA-U. Dnaj homolog subfamily B member 1 (DNAJB1) was decreased in JIA-U compared to I-CAU. The relevance of the other proteins is unknown (Fig. 2). These observations suggest that tear biomarker profiles could be useful in distinguishing subtypes of anterior uveitis.

### Network analyses

Gene ontology annotation using DAVID categorized pathways of molecular functions, cellular components, and biological processes. Ontology revealed 15 out of 29 altered proteins in pathways related to extracellular exosomes (*p =* 6.4 x 10^–6^) in both groups.

## Discussion

We demonstrated that tears, an easily obtained biospecimen, can detect biomarkers previously found to be present in AqH in children with uveitis. Furthermore, we found potentially novel cytokines, chemokines, and proteins in tears differentially expressed in JIA-U compared to I-CAU, suggesting intrinsic biologic differences, despite similar clinical phenotypes. While these findings require replication, they suggest that tears have a similar profile to AqH and may be useful in biomarker studies, and that there may be pathophysiologic differences between JIA-U and I-CAU.

AqH is secreted by the ciliary body and circulates in the anterior chamber of the eye, where inflammatory cells appear. Studies of AqH in adults and children with uveitis have shown correlations between certain cytokines (IL-6, IL-8 IL-10, IFN-γ, sVCAM, RANTES, and IP-10) and uveitis activity and between others (IL-1β, IL-2, IL-6, IL-8, IL-10, tumor necrosis factor-α, and vascular endothelial growth factor) and systemic immunosuppressive treatment [10, 17–19]. AqH therefore appears to reflect underlying uveitis activity; however, AqH collection is invasive and thus not feasible in children with JIA alone or when ocular surgery is not otherwise indicated. Tears are already utilized for biomarker discovery in systemic diseases with and without ocular involvement, such as Sjögren syndrome, RA, thyroid ophthalmopathy, malignancy, multiple sclerosis, and diabetes mellitus [13–15, 20–24]. If tears can reflect uveitis activity, similar to AqH, it may be a promising bios- pecimen for uveitis biomarker studies. Carreon and col- leagues report differences in the cytokine and chemokine tear profiles of adult uveitis patients compared to healthy controls and differences based on uveitis anatomic classification, wherein patients with anterior and panuveitis had increased concen- trations compared to controls and intermediate and posterior uveitis [25].

We confirmed the presence of sCD14, S100A8/A9, SAA1, LAP3, and TTR in tears, which have been identi- fied in AqH and serum of children with JIA-U (Table 2) [8–10, 12, 26]. There were differences in the level of ex- pression based on underlying uveitis diagnosis, but the relevance of their presence needs further exploration, as these cytokines are non-specific and are present in the serum of individuals with various autoimmune diseases. Nevertheless, this preliminary work supports the prem- ise that tears may also be used in biomarker studies in children at risk for developing de novo eye disease.

One prior study examined tears for biomarkers in 13 patients with JIA-U and 3 controls using high-resolution MS and report 236 proteins as candidate biomarkers for JIA-U [27]. While these specific findings were not repli- cated in our study, our comparison groups differed (i.e., normal controls were not used as a comparison group in our study).

We noted 29 unique proteins in the tear profiles of children with JIA-U and I-CAU. SAA1, HEXB, TIMP1, and ERN1 had increased expression in JIA-U and have been reported in patients with RA and JIA. They play a role in activation of fibroblast-like synoviocytes (FLS), immune modulation, angiogenesis, apoptosis, and inva- sive cell migration, where they are important in inflam- matory response and tissue injury or as markers of disease activity [28–34]. SAA1 is a major acute phase protein expressed in response to inflammation and tissue injury in patients with RA [28–30]. HEXB with co- factor GM2 activator protein catalyze the degradation of the ganglioside GM2 and other molecules containing terminal *N*-acetyl hexosamines in patients with RA [31]. TIMPs were increased in the plasma of untreated children with JIA, have been associated with levels of matrix metalloproteinase (MMP)-1, MMP-3, and TIMP1 in paired serum and synovial fluid also in those with JIA, and were found in AqH of patients with glaucoma [32, 33, 35]. DNAJB1 had low expression in children with JIA-U compared to those with I-CAU and has been associated with JIA [36]. In our study, proteins associated with arthritis were increased in the tear profile of children with JIA-U. There were other proteins of uncer- tain biological relevance that differed between the groups. Further study is needed in larger cohorts to determine their importance in uveitis. Our findings also show value in children with JIA, as we were able to detect arthritis-related markers.

Pathway analyses revealed that detected proteins were involved in extracellular exosomes. This pathway is related to proteins released from vesicles into the extracellular region by fusion of the limiting endosomal membrane of a multivesicular body with the plasma membrane. Because tears are typically acellular, these findings suggest that proteins may be secreted from cells possibly having differential gene expression related to underlying uveitis activity. Further study into the role of extracellular exosomes in the pathogenesis of uveitis and the cells secreting these proteins in children with uveitis should be investigated.

### Strengths and limitations

This uncontrolled pilot study included only a small number of children as a proof-of-principle that tears can identify proteins found in AqH. A limitation is that the sample size was too small to adjust for the potential confounders of treatment effect and level of disease activity, so these results should be considered as hypothesis-generating (Table 3). Differences in protein, cytokine, and chemokine levels have been reported in studies that examined AqH of children with uveitis and tears of adults with uveitis compared to controls without uveitis. However, it is important to examine the tear profile of children with JIA without uveitis and pediatric healthy controls without ocular disease since the controls in AqH studies had congenital cataracts and glaucoma. Serial tear collection with longitudinal follow-up is ongoing in children with JIA, other forms of uveitis, and pediatric controls to replicate and extend these findings to a larger group of study participants. These future studies will allow further comparisons by disease group, uveitis activity, and treatment response. A small number of children were included in our study, and it is not a reflection of the number of children diagnosed with uveitis in our practice. This was a pilot study to determine if we could detect proteins in tears that were previously detected in AqH and to refine our processes and methods. Not all children contributed tears from both eyes, as only the eyes with uveitis were included. We were not able to include all samples in our analysis due to issues with protein recovery which may be related to sample collection. Also, analyses did not adjust for correlation between the eyes of patients with bilateral disease. A strength of this study is that it is a pediatric study using state-of-the art techniques and demonstrates the feasibility of tear sample collection in this population.

**Table 3:** Characteristics of children with chronic anterior uveitis

Patient		Diagnosis, laterality	Age at diagnosis, years	Uveitis Active	Number of eyes included	Topical meds at time of collection	Systemic meds at time of collection
1	19-year-old NH AA female	Oligoarticular JIA-associated uveitis, unilateral	12	No AC cells rare OD; None OS	1	Prednisolone acetate every hour OD	Methotrexate oral
2	15-year-old NH white female	Oligoarticular JIA-associated uveitis, bilateral	1	No AC cells 0 OU	2	None	Adalimumab injections
3	17-year-old NHW female	Polyarticular rheumatoid factor negative JIA-associated uveitis, bilateral	5	No AC cells 0 OU	1	None	Infliximab infusions
4	15-year-old NHW female	Idiopathic CAU, bilateral	3	No AC cells O OU	1	None	Mycophenolate oral
5	17-year-old NH AA female	Idiopathic CAU, bilateral	15	Yes AC cells 1+ OU	2	Difluprednate 1 drop daily OU	Methotrexate injections
6	14-year-old NH AA female	Idiopathic CAU, bilateral	11	Yes AC cells 1+ OU	1	Difluprednate 1 drop daily OU	Methotrexate injections
7	12-year-old NH AA male	Idiopathic CAU, bilateral	6	No AC cells 0 OU	1	Prednisolone acetate 1 drop 2 times per day OS and Timolol maleate	Prednisolone acetate 1 drop 2 times per day OS and Timolol maleate

*JIA-U* JIA-associated uveitis, *CAU* chronic anterior uveitis, *NH* non-Hispanic, *AA* African-American, *W* White, *AC* anterior chamber, *OD* right eye, *OS* left eye, *OU*

bilateral eyes

## Conclusions

We demonstrated the utility of tears for biomarker studies in children with JIA and uveitis. The preliminary evidence of proteomic differences between JIA-U and I-CAU related to inflammatory arthritis generate hypotheses that warrant further investigation. Tear collection is well tolerated in children and has likely application in JIA studies. The use of tears in biomarker studies may improve our understanding of the underlying mechanisms involved in ocular inflammation which could lead to discovery of biomarkers for the early detection of uveitis and better prediction models for susceptibility in children with JIA, monitoring of disease, and treatment response.

## Methods

We performed a cross-sectional study approved by the Emory University Institutional Review Board, which con- formed to the US Health Insurance Portability and Privacy Act requirements. Informed consent/assent was obtained from parents and children accordingly. We followed the tenets of the Declaration of Helsinki.

### Subjects

Children with JIA-U and I-CAU were screened and enrolled at the Emory Eye Center during their routine ophthalmology clinic visits from October 2015 to March 2017. *Inclusion criteria* were (1) a diagnosis of chronic anterior uveitis diagnosed by a uveitis fellowship-trained ophthalmologist, with or without JIA by the International League of Associations for Rheumatology classification in children with arthritis [37] and (2) 5 years of age or older.

### Data collection

Data collected by medical record review included date of birth, sex, self-reported race/ethnicity, JIA category, uve- itis characteristics (onset date, diagnosis date, laterality, ocular complications), anterior chamber (AC) cell score per standardization of uveitis nomenclature (SUN) cri- teria [38], and ANA status. Use of topical and systemic medications was reviewed at time of tear collection. Data from the ophthalmic exam were recorded at time closest to tear collection.

### Tear collection

Ophthalmologists or trained study staff collected tear samples from children using Schirmer strips which are routinely used for dry eye evaluation. We included eyes with a history of uveitis only. After local anesthesia (topical 0.5% proparacaine hydrochloride, Bausch & Lomb, Roches- ter, NY, USA) was administered, residual anesthetic fluid was removed from the conjunctival cul-de-sac while avoiding corneal contact. A Schirmer strip was placed into the temporal inferior fornix of each eye (approximately 6 mm from the lateral canthus), avoiding the corneal surface. Reflex tearing was avoided as much as possible. The eye was closed for 5 min. Alternatively, the strip was removed when fully saturated (maximum 5 min). The Schirmer strip was placed in an Eppendorf micro centrifuge tube on ice and stored at − 80 °C until processing.

### Schirmer strip protein extraction

Each strip was soaked and vortexed in 500 uL of urea lysis buffer (8 M urea, 100 mM NaH2PO4, pH 8.5), including 5 uL (100× stock) HALT protease and phosphatase inhibitor cocktail (Pierce). Protein supernatants were transferred to 1.5-mL Eppendorf tubes, and proteins were extracted as previously published [39]. An aliquot equivalent to 10 μg of total protein was removed from each sample and combined to obtain two global internal standards (GIS) used later for tandem mass tag (TMT) labeling (total was split into two aliquots of 40 μg of total protein). For each sample, 40 μg of total protein was processed.

### TMT labeling

The samples were randomized over four TMT 10-plex batches. In each batch, the GIS samples took up channels 1 and 10 (TMT-126 and TMT-131, respectively). Labeling was performed according to the manufacturer’s protocol, and cleanup was performed according to a previously published method [39].

### ERLIC fractionation

The protocol for electrostatic repulsion interaction chro- matography (ERLIC) fractionation was adapted from a published method [40].

### LC-MS/MS analysis with MS3 quantitation

Liquid chromatography-tandem mass spectrometry with MS3 selective precursor selection (LC-MS/MS/SPS-MS3) was used to identify proteins in patients with JIA-U (*n* = 3) and I-CAU (*n* = 4). LC-MS/MS/SPS-MS3 was adapted from a published procedure [39, 41].

### Database search and TMT quantitation

MS/MS spectra were searched against a Uniprot curated human database (downloaded on 4/15/2015 with 90,300 sequences) with Proteome Discoverer 2.1 (ThermoFisher Scientific, San Jose, CA, USA). Search parameters and protein quantitation using MS3 reporter ions were previ- ously reported [39]. Ratio of sample over the GIS of nor- malized channel abundances were used for comparison across all samples.

### Statistical analysis

Descriptive statistics were performed using frequencies, percentages, medians, and IQR, as appropriate.

Protein expression values were analyzed using algorithms available in GeneSpring GX 13.0 (Agilent Technologies Inc., Santa Clara, CA, USA). Differential expression values were identified using Mann-Whitney *U* tests and presented as a heatmap. Hierarchical clustering using complete linkage was used to group proteins and samples by expression patterns.

Network analysis explored associations among proteins involved in different biological processes to find the most significant shared pathways using DAVID 6.7 (the Database for Annotation, Visualization and Integration Discovery) [42, 43]. The input data for the network analyses consisted of the significantly expressed proteins represented on the heatmap analysis.

Statistical significance was defined as two-sided *p* < 0.05. Each tear sample was treated independently. All analyses were conducted using SAS v. 9.4 for Windows (Cary, NC, USA).

## Additional file

Additional file 1: Cytokines and chemokines reported in pediatric uveitis biomarker studies using aqueous humor. (DOCX 13 kb)

## Abbreviations

AC: Anterior chamber
ANA: Antinuclear antibody
AqH: Aqueous humor
CAU: Chronic anterior uveitis
DAVID: The Database for Annotation, Visualization and Integration Discovery
DNAJB1: Dnaj homolog subfamily B member 1
ERLIC: Electrostatic repulsion interaction chromatography
ERN1: Serine/threonine-protein kinase/endoribonuclease IRE1
FLS: Fibroblast-like synoviocytes
GIS: Global internal standards
HEXB: Beta-hexosaminidase subunit beta
I-CAU: Idiopathic chronic anterior uveitis
IL-29/IFN-λ1: Interleukin-29/interferon-λ1
JIA-U: JIA-associated euveitis
JIA: Juvenile idiopathic arthritis
LAP3: Latency-associated peptide
LC-MS/MS: Liquid chromatography-tandem mass spectrometry
LC-MS/MS/SPS-MS3: Liquid chromatography-tandem mass spectrometry with synchronous precursor- based selection
MIF: Macrophage migration inhibitory factor
MMP: Matrix metalloproteinase
RA: Rheumatoid arthritis
RF: Rheumatoid factor
S100: S100 calcium-binding protein
SAA1: Serum amyloid A
SUN: Standardization of uveitis nomenclature
TIMP1: Metalloproteinase inhibitor 1
TMT: Tandem mass tag
TTR: Transthyretin
