# Supplementary material for: Discovery of tear biomarkers in children with chronic non-infectious anterior uveitis: a pilot study
Source: J Ophthalmic Inflamm Infect. 2018 Oct 16;8:17. doi: 10.1186/s12348-018-0156-5 (PMC6191408; doi:10.1186/s12348-018-0156-5)
Supplement: Supplementary file 1 — Cytokines and chemokines reported in pediatric uveitis biomarker studies using aqueous humor. (DOCX 13 kb) [file 12348_2018_156_MOESM1_ESM.docx]

Additional file 1: Appendix 1.

Cytokines and chemokines reported in pediatric uveitis biomarker studies using aqueous humor.

Angiotensin converting enzyme (ACE), amphiregulin, angiopoietin (Ang)-2, brain derived neutrotrophic factor (BDNF), B lymphocyte chemoattractant (BLC), complement component 5a (C5a), chemokine (CCL) 5, cluster of differentiation (CD) 106, eotaxin/CCL11, ICAM-1, interferon (IFN) gamma, interleukin (IL)-2, IL-5, IL-6, IL-8/ chemokine (CXCL) 8, Il-10, IL-13, IL-15, IL-18, IL-22, IL-26, IL-29, interferon gamma-induced protein (IP)-10/CXCL10, latency associated peptide (LAP), monocyte chemoattractant protein (MCP)-1/CCL2, macrophage migration inhibitory factor (MIF), monokine induced by gamma interferon (MIG)/CXCL9, macrophage inflammatory protein (MIP)-3βCCL19, macrophage-derived cytokine (MDC)/CCL22, nerve growth factor (NGF), osteoprotegerin (OPG), regulated on activation normal T-cell expressed and secred (RANTES), serum amyloid A (SAA-1), soluble intercellular adhesion molecule 1 (sICAM1), soluble IL-2 receptor, (sIL-2R), soluble vascular cell adhesion protein (sVCAM), S100 calcium binding protein (S100) A8/A9, A12, tyrosine kinase with immunoglobulin-like and EGF-like domains (TIE-2)/ tunica endothelial kinase (TEK), TNF alpha, transthyretin (TTR), and vascular endothelial growth factor (VEGF).
